# Supplementary material for: Interpretation of continuously measured vital signs data of COVID-19 patients by nurses and physicians at the general ward: A mixed methods study
Source: PLoS One. 2023 May 25;18(5):e0286080. doi: 10.1371/journal.pone.0286080 (PMC10212076; doi:10.1371/journal.pone.0286080)
Supplement: S1 Table — Total number of participants is 24. (DOCX) [file pone.0286080.s001.docx]

| Supplemental table 1. Number of participants giving a certain answer, per case. Total number of participants is 24. | | | | | | | | | | | | | | | | | | | | | |
| --- | --- | --- | --- | --- | --- | --- | --- | --- | --- | --- | --- | --- | --- | --- | --- | --- | --- | --- | --- | --- | --- |
| **Case number:** | | **1** | **2** | **3** | **4** | **5** | **6** | **7** | **8** | **9** | **10** | **11** | **12** | **13** | **14** | **15** | **16** | **17** | **18** | **19** | **20** |
| **Question** | **Answer** |  |  |  |  |  |  |  |  |  |  |  |  |  |  |  |  |  |  |  |  |
| Situation? | - Improving | 0 | 3 | 4 | 11 | 0 | 2 | 0 | 0 | 9 | 1 | 2 | 1 | 0 | 5 | 0 | 8 | 2 | 2 | 0 | 8 |
|  | - Stable | 11 | 14 | 14 | 12 | 1 | 7 | 15 | 12 | 15 | 17 | 20 | 1 | 16 | 19 | 7 | 13 | 14 | 22 | 2 | 16 |
|  | - Deteriorating | 13 | 7 | 6 | 1 | 23 | 15 | 9 | 12 | 0 | 6 | 2 | 22 | 8 | 0 | 17 | 3 | 8 | 0 | 22 | 0 |
|  |  |  |  |  |  |  |  |  |  |  |  |  |  |  |  |  |  |  |  |  |  |
| Action required? | Yes | 21 | 5 | 5 | 3 | 22 | 16 | 13 | 16 | 1 | 7 | 7 | 23 | 11 | 5 | 20 | 5 | 10 | 2 | 24 | 5 |
|  |  |  |  |  |  |  |  |  |  |  |  |  |  |  |  |  |  |  |  |  |  |
| Expected respiratory insufficiency? | Yes | 18 | 3 | 1 | 0 | 20 | 20 | 6 | 17 | 1 | 0 | 0 | 22 | 3 | 0 | 5 | 2 | 2 | 0 | 21 | 0 |
|  |  |  |  |  |  |  |  |  |  |  |  |  |  |  |  |  |  |  |  |  |  |
| *Was respiratory insufficiency observed** | | yes | yes | no | no | yes | yes | no | yes | yes | no | no | yes | no | yes | no | no | yes | no | yes | no |
|  |  |  |  |  |  |  |  |  |  |  |  |  |  |  |  |  |  |  |  |  |  |
| Difficult case? | Yes | 15 | 10 | 4 | 5 | 7 | 14 | 8 | 13 | 2 | 7 | 3 | 5 | 6 | 2 | 7 | 7 | 14 | 7 | 12 | 2 |
| *The actual observed occurrence of the outcome respiratory insuffiency in the hours following the data as shown to participants. | | | | | | | | | | | | | | | | | | | | | |
