# Supplementary material for: Interpretation of continuously measured vital signs data of COVID-19 patients by nurses and physicians at the general ward: A mixed methods study
Source: PLoS One. 2023 May 25;18(5):e0286080. doi: 10.1371/journal.pone.0286080 (PMC10212076; doi:10.1371/journal.pone.0286080)
Supplement: S1 File — (DOCX) [file pone.0286080.s002.docx]

Supplemental material 1. Semi structured interview guide (translated from Dutch)

- You just did a number of case reviews. Can you explain how you tackled such a case review?
- What did you take into account during the review? If not mentioned, ask after the use of: timeframe length, context information, starting and endpoint, coherence
- What influenced your decision to take action?
- What influenced your estimation of respiratory insufficiency?
- Why did you consider case X difficult?
- Why did you consider case X easy?
- Did you miss information to make a good estimation? What information did you miss?
